# Decrease in Cerebral Blood Flow after Reoxygenation Is Associated with Neurological Syndrome Sequelae and Blood Pressure

**DOI:** 10.3390/brainsci13111600

**Published:** 2023-11-17

**Authors:** Yanqiu Liu, Fengjuan Yuan, Zhongwei Peng, Yadong Zhan, Jianzhong Lin, Ran Zhang, Jiaxing Zhang

**Affiliations:** 1 Institute of Brain Diseases and Cognition, School of Medicine, Xiamen University, Xiamen 361102, China; 2 Fujian Provincial Key Laboratory of Neurodegenerative Disease and Aging Research, Xiamen University, Xiamen 361102, China; 3 Department of Neurology, Zhongshan Hospital of Xiamen University, School of Medicine, Xiamen University, Xiamen 361004, China; 4 Department of Radiology, Zhongshan Hospital of Xiamen University, School of Medicine, Xiamen University, Xiamen 361004, China

**Keywords:** CBF, ASL, HA, hypoxia, reoxygenation

## Abstract

Changes in cerebral blood flow (CBF) and regulation of cerebral circulation occur at high altitude (HA). However, the changes in CBF and their associations with neurological syndrome sequelae and blood pressure after subjects return to the lowlands remain unclear. In this study, the subjects were 23 college students who were teaching at an altitude of 4300 m for 30 days. These subjects were studied before reaching the HA (Test 1), one week after returning to the lowlands (Test 2), and three months after returning to the lowlands (Test 3). Symptom scores for de-acclimatization syndrome were evaluated. Changes in CBF were measured using the magnetic resonance imaging arterial spin labeling (ASL) technique. Additionally, the velocity of CBF in the cerebral arteries was measured using a transcranial doppler (TCD). In Test 2 vs. Test 1, the peak systolic velocity and mean velocity in the basilar artery were significantly decreased. CBF exhibited significant decreases in the left putamen/cerebellum crus1/vermis and right thalamus/inferior temporal gyrus, while significant increases were observed in the left postcentral gyrus/precuneus and right middle cingulate gyrus/superior frontal gyrus. In Test 3 vs. Test 1, the basilar artery velocity returned to the baseline level, while CBF continued to decrease. The mean global CBF showed a decreasing trend from Test 1 to Test 3. Furthermore, the mean global CBF had a negative correlation with the systolic pressure, pulse pressure, and mean arterial pressure. The decrease in CBF after reoxygenation may underlie the neurological symptoms in subjects returning to the lowlands. Increased blood pressure could serve as a predictor of a decrease in CBF.

## 1. Introduction

At a high altitude (HA), hypoxia is a potent vasodilator which can oppose the vasoconstriction induced by hyperventilation, leading to an increase in cerebral blood flow (CBF). An increase in CBF could reduce the severity of central sleep apnea at HA [1]. However, in most cases, an altered CBF may be associated with HA diseases. Those with acute mountain sickness (AMS) have higher CBF than those without AMS [2,3,4,5,6]. In contrast, in the Qinghai–Tibetan Plateau, Han immigrants are associated with HA diseases. Those with chronic mountain sickness (CMS) show lower CBF, and patients with neurological deterioration show more disorganized CBF [7]. Some studies have suggested that impaired regulation of the cerebral circulation may serve as a potential pathogenesis for AMS [8,9]. A number of studies have reported that the dynamic cerebral pressure–flow relationship is impaired under conditions of hypobaric hypoxia [10].

In one study, the CBF velocity increased in the anterior and middle cerebral arteries at 3440 m, but with further ascent to 5050 m, this increase was followed by a decrease. However, the CBF in the posterior cerebral artery continued decreasing during the entire expedition, from an altitude of 3440 m to 5050 m [2]. After the subjects acclimatized to HA, the CBF velocity returned to the baseline level tested at sea level. The velocities of CBF in the internal carotid artery and vertebral artery were slightly increased after subjects stayed at Pikes Peak (4300 m) for between 18 and 44 h, and returned to sea level values 4 days later [11]. The CBF velocity in the middle cerebral artery increased immediately, and then normalized after subjects stayed at 5260 m for 30 days [12]. The CBF velocity returned to sea level values after the subjects stayed at 5260 m for 3 weeks [13]. In summary, CBF increases during acute HA exposure, followed by a decrease during long-term stays at HA, and the increased CBF tends to return to near sea level values after 3–5 days of acclimatization [14,15].

When HA residents move to lowlands, the relatively higher air oxygen increases their brain oxygen concentration, and the altitude-acclimatized brain is likely to suffer from oxidative stress generated by reoxygenation. During the initial stage, HA de-acclimatization reactions occur [16]. Animal studies have shown that CBF decreases immediately after reoxygenation. For example, in newborn pigs, the blood flow in the carotid artery was significantly decreased compared to the baseline level after 4 h of reoxygenation [17]. In rats, CBF increased during acute hypoxia and returned to the baseline immediately after reoxygenation [18]. In mice undergoing hypoxic–ischemic insult, during the reperfusion phase, the mean CBF level gradually rose, nearly returning to its pre-surgery level after 9 h of reperfusion, and then decreased [19]. In healthy subjects, CBF was found to decrease in deep nuclei during reoxygenation [20]. In patients with traumatic brain injury, a reduction in CBF was observed in the concussive hypoxic regions [21].

Previous studies have demonstrated that HA de-acclimatization is associated with neurological and psychological symptoms [22,23]. In the present study, we hypothesized that CBF could be reduced after subjects returned to lowlands from HA, and the decrease in CBF may be associated with neurological syndrome sequelae during the de-acclimatization phase. Therefore, lowland college students who volunteered for 30 days teaching on the Qinghai–Tibet plateau were studied. Previous studies concerning CBF at HA have mainly been measured using transcranial doppler (TCD) ultrasound [2,6]. In this study, changes in CBF were measured using arterial spin labeling (ASL) based on magnetic resonance imaging (MRI), which does not require exogenous contrast agents; instead, it uses magnetically labeled arterial blood water as an endogenous tracer and easily allows voxel-by-voxel quantification of brain perfusion [24].

## 2. Materials and Methods

### 2.1. Participants

The subjects were 23 healthy college students (12 males and 11 females, aged 19.4 ± 0.9 years). All subjects had a normal body mass index and volunteered as teachers on the Qinghai–Tibet plateau for a period of 30 days. They had been born and were living in the lowlands (<500 m). During their stay on the plateau, no supplementary oxygen was provided to any of the subjects. Subjects with a history of head injuries or neurological disorders were excluded from the study. None of the subjects were smokers. The procedure was explained to all subjects. This study was conducted in accordance with the Declaration of Helsinki, and the Research Ethics Review Board of Xiamen University approved this experimental protocol (XDYX2016013).

### 2.2. HA Exposure and Experimental Design

A previous study showed that HA has a range of pathophysiological effects on the brain that can usually be reduced by slowing the rate of ascent [25]. Therefore, in order to acclimate to the HA environment and avoid AMS, these subjects chose a slower train as their form of transportation to the plateau. All subjects spent 3 days traveling by train to Lhasa (3650 m), Tibet, China. Four days later, the subjects spent four hours traveling to Dangxiong (4300 m), Tibet. After their 30-day stay at the plateau, they descended to Lhasa again. Four days later, these subjects also spent three days returning to the lowlands by train. At Dangxiong, subjects consumed similar food and drink to what they had at Xiamen. No subject was allowed to drink. A baseline set of tests was initially conducted before ascent to HA (Test 1), and the same set of tests were performed one week (Test 2) and three months (Test 3) after subjects returned to the lowlands, respectively. A previous study found that people can adapt to an HA environment after 30 days, and it takes about 20 days to adapt to the lowlands after returning from HA [26,27]. In this study, we focused on the changes after reoxygenation, so we chose Test 1 as the baseline and Test 2 as the time point at which subjects were not fully adapted to the lowlands after reoxygenation. In addition, we took into account the fact that various changes caused by HA may require a longer reoxygenation time, so we chose Test 3 as the time point at which subjects were fully adapted to the lowlands after reoxygenation.

### 2.3. Physiological Measurements

Blood pressure, heart rate, arterial oxygen saturation (SaO_2_), erythrocyte, hematocrit, hemoglobin, and pulmonary function were measured. Blood samples were collected in the morning, between 07:00 and 07:30.

### 2.4. Evaluations of Mountain Sickness and De-Acclimatization Symptoms

The Lake Louise scores were measured in the morning, between 8:00 and 10:00, on the first day when subjects arrived at Dangxiong and on the thirtieth day after their stay in Dangxiong, respectively. The symptom score component consisted of five symptoms: headache, gastrointestinal discomfort, fatigue and weakness, dizziness, and sleep difficulties. A Lake Louise score greater than 4 was defined as AMS [28]. The de-acclimatization scale was tested using Test 2 and Test 3. The de-acclimatization scale was divided into neurological symptoms and other symptoms. Neurological symptoms included insomnia, fluster, dizziness, lethargy, vertigo, and responsiveness; other symptoms included an increase/loss of appetite, foreign body sensation in the pharynx, swelling of the body, asthma, fatigue, irregular pulse, and chest congestion. The symptom scores and diagnostic criteria for de-acclimatization symptoms were adopted from a previous study (no response, 0–4; mild, 5–14; moderate, 15–24; severe, >25) [29].

### 2.5. TCD Measurement

A TCD device (Doppler-Box, DWL Compumedics, Singen, Germany) operating at 2 MHz was used for our measurements. In all subjects, the anterior cerebral artery (ACA), middle cerebral artery (MCA), and basilar artery (BA) were measured through the temporal window at depths of 54 mm, 60 mm, and 90 mm, respectively. The tested cerebral hemodynamic parameters included peak systolic velocity (Vs), end-diastolic velocity (Vd), mean velocity (Vm), pulsatility index (PI), and resistance index (RI). TCD was performed in a horizontal position with the subject awake.

### 2.6. MRI Acquisition

Brain images were acquired using a Tim Trio 3T scanner (Siemens, Erlangen, Germany) at the MRI Center in Zhongshan Hospital of Xiamen University. A 3D structural MRI was obtained using a T1-weighted MPRAGE sequence (TR/TE = 1900/2.7 ms, FOV = 250 × 250 mm^2^, matrix = 256 × 256, flip angle = 9°, and slice thickness = 1 mm). CBF images were obtained using an echo-planar imaging (EPI) sequence with the following parameters: TR/TE = 3000/14 ms, FOV = 192 × 192 mm^2^, matrix = 128 × 128, flip angle = 90°, and slice thickness = 4.5 mm.

### 2.7. Voxel-Based Analysis of Group CBF Difference

Dcm2nii 0.9.0.1 software was used to convert the brain images from scanner format to NIFTI format. The data analysis process was described in our previous study [30]. The images were preprocessed using the SPM12 package (http://www.fil.ion.ucl.ac.uk/spm/, accessed on 19 January 2021). CBF was calculated using an ASL perfusion MRI data processing toolbox (ASLtbx). Images were first registered to the T1-weighted structural images, and then to the Neurological Institute (MNI) standard space after a stepwise registration within the framework of the FMRIB Software Library (FSL). Voxel-based analysis was conducted using Analysis of Functional Neuro-Images (AFNI_2011_12_21_1014) software (https://afni.nimh.nih.gov/). Subsequently, the CBF images were spatially smoothed with a Gaussian kernel of 6 mm full-width at half-maximum (FWHM). A paired sample *t*-test was used to detect the differences in CBF between Test 2 and Test 1, as well as between Test 3 and Test 1. Meanwhile, gender and age were used as covariates. The statistical parametric map was generated at *p* < 0.05 and corrected by Alphasim multiple comparisons.

### 2.8. Statistical Analysis

All data were analyzed using SPSS 19 software. For all data, paired sample *t*-tests were used for the comparison of Test 2 with Test 1 and of Test 3 with Test 1. Data are presented as the mean ± standard deviation (SD). Linear regression correlations were used to assess the correlation of mean global CBF with blood pressure and TCD blood velocity. Data were plotted using GraphPad Prism 7 software. For all analyses, *p* < 0.05 was considered significant.

## 3. Results

### 3.1. Scores of AMS and De-Acclimatization Symptoms

Compared with the first day after the subjects arrived at Dangxiong, the Lake Louise scores were significantly decreased on the 30th day that subjects had stayed at Dangxiong (*p* = 0.037) (Table 1).

**Figure 1:**
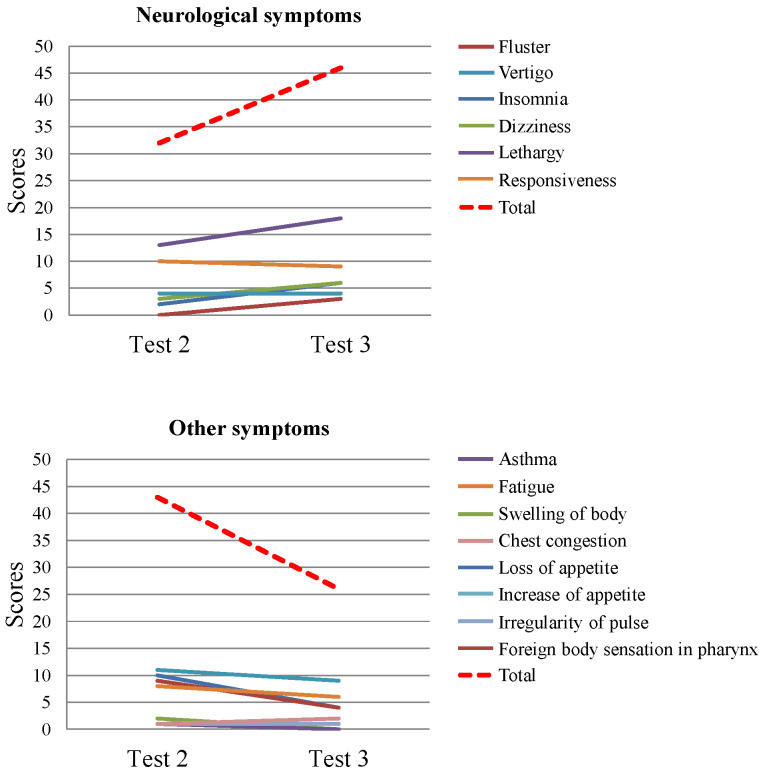
Scores of neurological symptoms and other symptoms according to Test 2 and Test 3. Dotted line indicates the total scores of neurological symptoms and other symptoms.

In Test 2, 15 subjects had neurological symptoms and 8 subjects had other symptoms. In Test 3, compared with Test 2, there was an increase in the total score for neurological symptoms and a decrease in the total scores for other symptoms (Figure 1).

### 3.2. Physiological Changes

In Test 2, compared with Test 1, the heart rate, erythrocyte, hemoglobin, and hematocrit were significantly increased (*p* = 0.027, *p* = 0.005, *p* = 0.001, and *p* = 0.008, respectively), while the forced expiratory volume in one second (FEV1) and FEV1/forced vital capacity (FVC) were significantly decreased (*p* = 0.039 and *p* = 0.020, respectively) (Table 2).

In Test 3, compared with Test 1, the heart rate, systolic pressure, diastolic pressure, pulse pressure, and mean arterial pressure were significantly increased (*p* = 0.042, *p* < 0.001, *p* = 0.012, *p* = 0.007, and *p* = 0.001, respectively) (Table 2).

### 3.3. CBF in Cerebral Arteries

**Figure 2:**
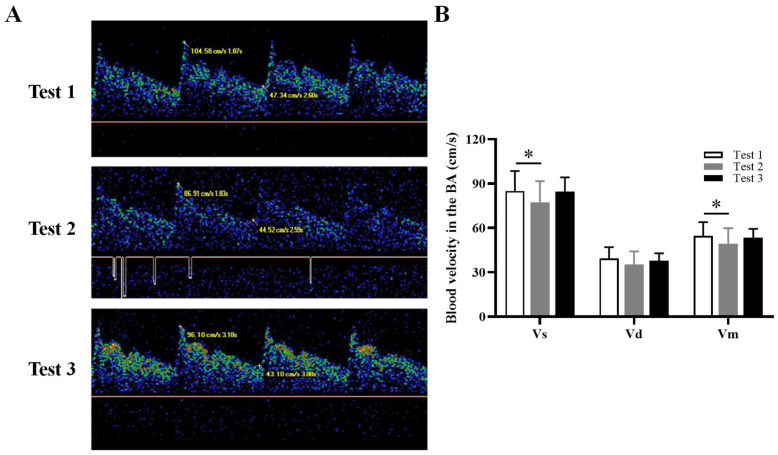
(**A**) Representative images of BA measurements according to Test 1, Test 2, and Test 3. (**B**) Quantification of blood velocity in the BA according to Test 1, Test 2, and Test 3. BA, basilar artery; Vs, peak systolic velocity; Vd, end-diastolic velocity; Vm, mean velocity. * *p* < 0.05.

The TCD measurements showed that blood Vs and Vm in the BA were significantly decreased in Test 2 compared with Test 1 (*p* = 0.028, *p* = 0.039, respectively) (Figure 2). There were no significant differences in the blood measurements of the ACA and MCA between Test 2 and Test 1, nor between Test 3 and Test 1 (Figure S1). In addition, there were no significant differences in the PI and RI values for ACA, MCA, and BA between Test 2 and Test 1, nor between Test 3 and Test 1 (Figure S2).

### 3.4. Regional CBF

**Figure 3:**
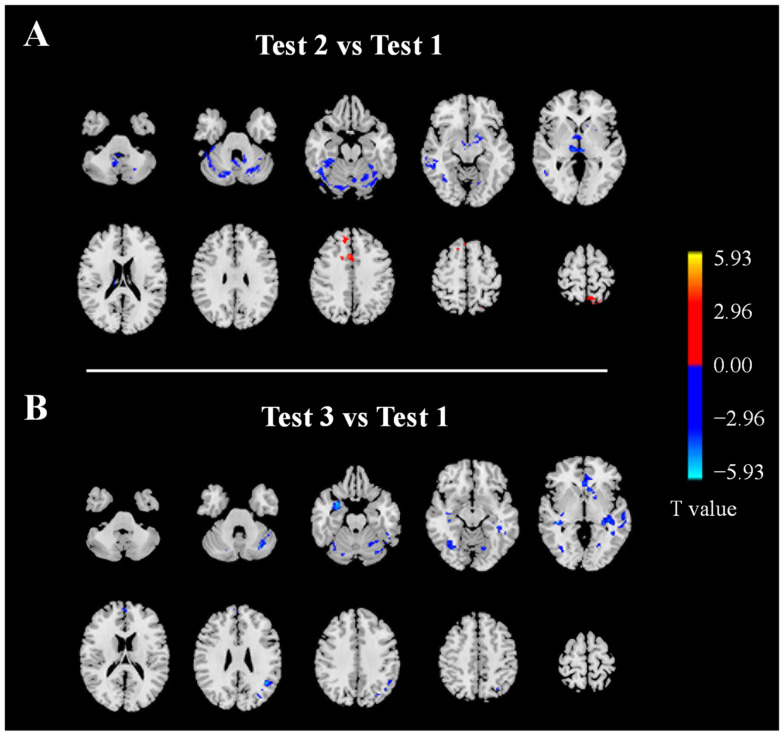
CBF changes in Test 2 (**A**) and Test 3 (**B**) compared with Test 1. Red indicates an increase and blue indicates a decrease.

In Test 2, compared with Test 1, the CBF was significantly decreased in the left putamen, left cerebelum_crus1/vermis, right thalamus, and right inferior temporal gyrus, while it was significantly increased in the left postcentral gyrus, left precuneus, right middle cingulate gyrus, and right superior frontal gyrus (Figure 3A, Table S1).

In Test 3, compared with Test 1, the CBF was significantly decreased in the left middle temporal gyrus, left lingual gyrus, left cerebelum_crus1, left parietal gyrus, left middle temporal gyrus, left cingulate gyrus, right amygdala, right hippocampus, right fusiform gyrus, and right cerebellum (Figure 3B, Table S1).

### 3.5. Mean Global CBF

**Figure 4:**
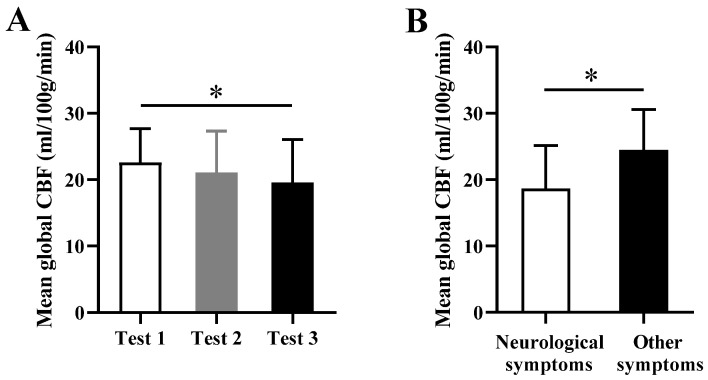
(**A**) Mean global CBF in Test 1, Test 2, and Test 3. (**B**) Mean global CBF values in the subjects with (n = 15) and without (n = 8) neurological symptoms in Test 2. * *p* < 0.05.

The mean global CBF showed a decreasing trend from Test 1 to Test 3, and there was a significant decrease in Test 3 compared with Test 1 (*p* = 0.041) (Figure 4A).

In Test 2, the mean global CBF value of subjects with neurological symptoms was significantly lower than that of subjects without neurological symptoms (*p* = 0.027) (Figure 4B).

### 3.6. Correlations

**Figure 5:**
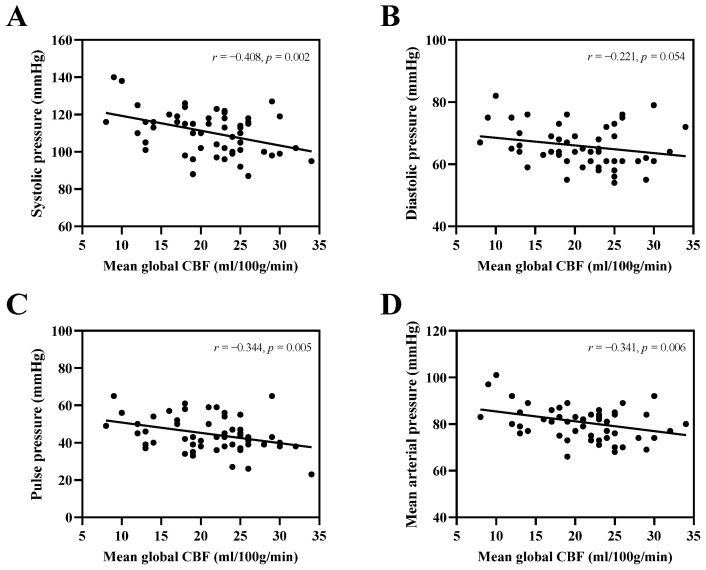
Correlation of mean global CBF with blood pressure. Correlations of mean global CBF with systolic pressure (**A**), diastolic pressure (**B**), pulse pressure (**C**), and mean arterial pressure (**D**), respectively.

The mean global CBF value had a significant negative correlation with systolic pressure (*r* = −0.408, *p* = 0.002), pulse pressure (*r* = −0.344, *p* = 0.005), and mean arterial pressure (*r* = −0.341, *p* = 0.006), respectively (Figure 5A,C,D). Moreover, the mean global CBF value had significant positive correlations with vs. (*r* = 0.248, *p* = 0.035), Vd (*r* = 0.261, *p* = 0.028), and Vm in the BA (*r* = 0.263, *p* = 0.028), respectively (Figure S3).

## 4. Discussion

The present study assessed the change in CBF and its correlations with neurological symptoms and blood pressure. Immediately after the subjects returned to sea level, blood velocity and regional CBF in the posterior brain significantly decreased. Three months after the subjects returned to sea level, the regional CBF continued to decrease, and the mean global CBF showed a decreasing trend over time at sea level. Interestingly, blood systolic pressure, pulse pressure, and mean arterial pressure all significantly increased and showed negative correlations with the mean CBF value. With the time spent living at sea-level, the neurological symptoms were aggravated; the mean global CBF value was significantly lower in subjects with neurological symptoms compared to those without.

Cerebellar impairment may be more severe than cortical impairment during the ascent to extreme altitudes [31]. Blood flow velocities in the BA were significantly reduced in extreme-altitude climbers compared to healthy controls [32]. A study using the TCD approach found that CBF values in the internal carotid artery and BA were lower in children living on the plateau than in children living at sea level, with whole-brain CBF values reduced by approximately 30% in children living on the plateau [33]. In the present study, we found a significant reduction in blood flow velocity in the BA after HA exposure, which was consistent with previous findings. However, there were no significant changes in the PI or RI values of the BA after HA exposure; these serve as indicators of the diastolic and resistance status of blood vessels. From this, we can infer that the decrease in blood flow velocity in the BA after exposure to HA was not due to an increase in the diameter of the blood vessels. Subsequently, the changes in CBF were investigated using the ASL method, and it was found that the brain regions with changes in CBF also occurred mainly in the posterior region of the brain, which was mutually confirmed by the results of TCD. Moreover, our study also found that the mean global CBF value had a significant, positive correlation with blood flow velocity in the BA. Therefore, we further deduced that the decrease in blood flow velocity in the BA after reoxygenation was due to a reduction in blood flow. In addition, hypoxia/reoxygenation-induced changes in CBF were also found in the posterior brain regions of patients with obstructive sleep apnea [34]. The brain regions with changes in CBF were consistent with those found in previous studies of brain structure and cellular EEG activity in plateau populations in our laboratory [35,36,37], suggesting that alterations in CBF may be a mechanism for structural changes in the brain.

A long-lasting decrease in CBF was first observed in this hypoxia/reoxygenation study. Previous studies had only observed a decrease in CBF shortly after reoxygenation [17,19]. The impaired regulation of cerebral circulation during hypoxic exposure and reoxygenation may potentially underlie this decrease in CBF [8,9]. In addition, when people descend to the lowlands after a period of HA living, the altitude-acclimatized brain is likely to suffer from the stresses generated by relatively high-level oxygen, which may disturb the constriction of blood vessels. Hyperoxia has been proven to cause a reduction in cerebral perfusion [38,39,40].

Moreover, there was an increase in the pulse pressure as a consequence of the greatly increasing systolic blood pressure and slightly increasing diastolic blood pressure three months after the subjects had returned to sea level. An increase in both systolic pressure and diastolic pressure with decreased CBF have been observed in many diseases. Patients with Type 2 diabetes mellitus exhibited decreased CBF, and both systolic pressure and diastolic pressure were inversely correlated with CBF [41]. Both regional and global resting CBF were decreased in hypertensive patients [42]. Acclimatization to HA hypoxia for 4 weeks has been shown to be accompanied by striking and long-lasting sympathetic overactivity 3 days after returning to sea level [23]. Thus, we speculate that the increases in systolic and diastolic blood pressure may be attributed to sympathetic overactivity.

Neurological symptom scores increased over time at sea level, suggesting increased mental fatigue or an impairment of the attention processes after hypoxia/reoxygenation. Our finding of decreased CBF in the anterior cingulate cortex, inferior parietal regions, and cerebellum may underlie the mechanism responsible for this. These regions have been proven to be associated with attention processes [43]. A study on mice undergoing hypoxic–ischemic insult showed that the degree of reduced CBF during reperfusion was well-correlated with the degree of later morphological brain damage [18]. A decrease in CBF has been confirmed to accompany hypoxic neurological syndromes. For example, reductions in CBF were found in subjects with vertigo [44], in patients with subacute mild traumatic brain injury (dizziness and simulator sickness) [21], in subjects after acute sleep restriction [45], and in participants with brain fatigue from a sustained mental workload [46]. A decrease in global CBF has also been shown to occur due to chronic hypoxia exposure. For example, Tibetans living on the Qinghai–Tibetan plateau exhibited significantly lower CBF compared to lowland Han subjects [30].

Our study has several limitations. First, we did not further confirm neuroimaging changes by excluding the effects of climate factors (low temperature, low pressure, strong solar radiation, etc.). Second, we did not use a longer follow-up period to clarify the time point of CBF recovery after 1 month of HA exposure. In addition, sleep, exercise, and diet at HA did not seem to play significant roles in causing changes in CBF. Subjects with a Lake Louise score of more than 4 were considered to have mountain sickness in HA. The Lake Louise Score section included an option for sleep difficulties, and it was found that none of the subjects experienced sleep difficulties at HA. The subjects had access to food at HA similar to what had been available in Xiamen, and there were no significant changes in their dietary habits. Furthermore, the main activity of these subjects in HA was teaching, and their exercise was similar to that in Xiamen.

In conclusion, both regional and global CBF continued to decrease over time after reoxygenation. Impaired regulation of the cerebral circulation may underlie the mechanism involved in CBF reduction. A decrease in CBF was found to underlie the neurological symptoms after the subjects returned to sea level. Increases in blood pressure may be attributed to sympathetic overactivity, and could serve as predictors of a decrease in CBF after reoxygenation. The decrease in CBF after returning to the sea level for three months suggests that recovery from brain damage takes a long time. Whether and for how long brain damage can be recovered from requires further investigation, and the underlying mechanisms still need to be studied in animal models.

## Figures and Tables

**Table 1:** Demographic characteristics of the subjects.

Characteristics	Subjects
Number of subjects	23
Gender (female/male)	11/12
Age (years)	19.4 ± 0.9
Education (years)	13.6 ± 0.9
BMI (kg/m^2^)	20.5 ± 1.9
Lake Louise score	
The first day at HA	1.7 ± 0.5
The thirtieth day at HA	0.48 ± 0.3
*p*-value	0.037

Data are presented as mean ± standard deviation (SD). BMI = body mass index; HA = high altitude.

**Table 2:** Physiological characteristics of the subjects.

Characteristics	Test 1	Test 2	Test 3	*p*1	*p*2
SaO_2_ (%)	98.3 ± 0.5	98.3 ± 0.5	98.1 ± 0.8	0.747	0.260
Heart rate (beats/min)	67.4 ± 10.8	72.5 ± 11.2	71.4 ± 8.2	0.027	0.042
Blood pressure (mmHg)					
Systolic pressure (mmHg)	107.2 ± 8.5	107.1 ± 11.8	117.4 ± 11.4	0.477	<0.001
Diastolic pressure (mmHg)	64.8 ± 6.4	63.1 ± 6.9	68.6 ± 5.6	0.109	0.012
Pulse pressure (mmHg)	42.4 ± 6.7	44.0 ± 10.2	48.8 ± 9.5	0.230	0.007
Mean arterial pressure (mmHg)	79.0 ± 6.5	77.8 ± 7.4	84.9 ± 6.6	0.218	0.001
Hematological measurements					
Erythrocyte (10^12^/L)	4.9 ± 0.5	5.1 ± 0.8	4.7 ± 0.7	0.005	0.494
Hemoglobin (g/L)	130.5 ± 13.3	140.1 ± 17.1	132 ± 12.8	0.001	0.400
Hematocrit (%)	40.2 ± 3.1	42.7 ± 4.3	39.7 ± 3.1	0.008	0.556
Pulmonary functions					
FVC (mL)	3609.7 ± 686.1	3664.7 ± 727.9	3681.4 ± 765.1	0.544	0.341
FEV1 (mL)	3168.7 ± 519.5	3014.3 ± 570.4	3059.9 ± 582.3	0.039	0.293
FEV1/FVC (%)	89.0 ± 11.2	83.5 ± 12.7	84.1 ± 10.6	0.020	0.054

Data are presented as mean ± standard deviation (SD). SaO_2_ = arterial oxygen saturation; FVC = forced vital capacity; FEV1 = forced expiratory volume in one second; *p*1: Test 2 vs. Test 1; *p*2: Test 3 vs. Test 1.

## Data Availability

The datasets used and/or analyzed during the current study are available from the corresponding author upon reasonable request.

## Supplementary Materials

The following supporting information can be downloaded at: https://www.mdpi.com/article/10.3390/brainsci13111600/s1.
