# Supplementary material for: Decrease in Cerebral Blood Flow after Reoxygenation Is Associated with Neurological Syndrome Sequelae and Blood Pressure
Source: Brain Sci. 2023 Nov 17;13(11):1600. doi: 10.3390/brainsci13111600 (PMC10669967; doi:10.3390/brainsci13111600)
Supplement: Supplementary file 1 [file brainsci-13-01600-s001.zip › brainsci-2663206-supplementary.pdf]

Figure S1. Quantification of blood velocity in the ACA and MCA in Test 1, Test 2, and Test 3. ACA, anterior cerebral artery; MCA, middle cerebral artery; Vs, peak systolic velocity; Vd, end-diastolic velocity; Vm, mean velocity.

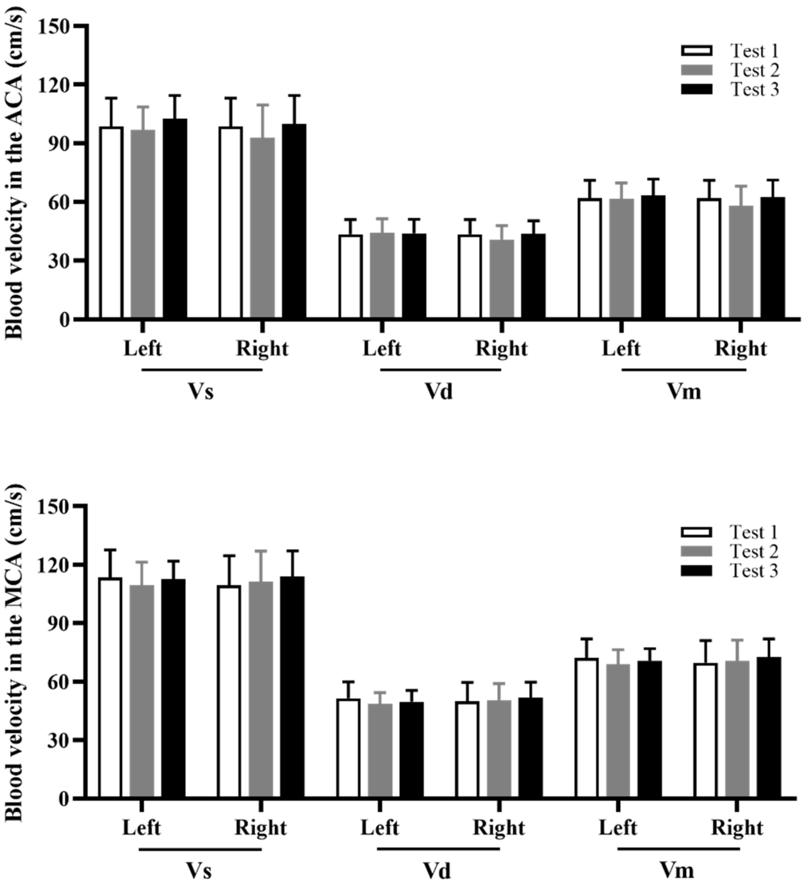

Figure S2. Quantification of PI and RI in the BA, ACA, and MCA in Test 1, Test 2, and Test 3. PI, pulsatility index; RI, resistance index; BA, basilar artery; ACA, anterior cerebral artery; MCA, middle cerebral artery.

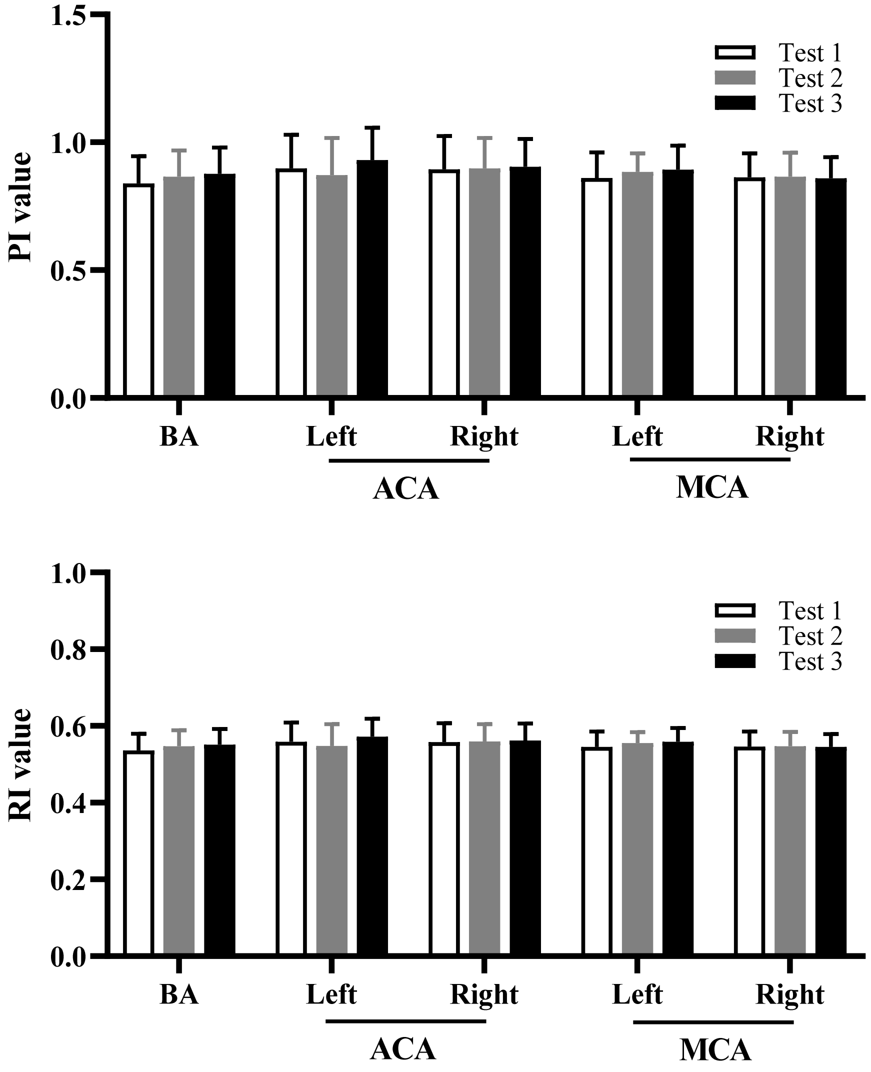

Figure S3. Correlation of mean global CBF with Vs, Vd, and Vm in the BA, respectively. CBF, cerebral blood flow; Vs, peak systolic velocity; Vd, end-diastolic velocity; Vm, mean velocity; BA, basilar artery.

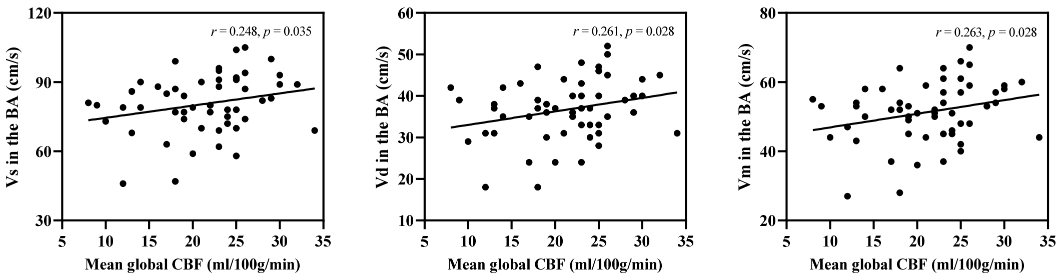

Table S1. Regional information of changed CBF in Test 2 and Test 3 compared with Test 1.

**Areas**	**Volume (voxels)**	**MNI coordinate**			***t* (peak)**
		x	y	z	
**Test 2 vs Test 1**					
Left postcentral gyrus	114	-7	-42	72	3.7
Left cerebelum_vermis	124	-2	-46	-34	-3.93
Left precuneus	149	-5	-66	64	3.66
Left putamen	374	-16	0	-8	-4.39
Left cerebelum_crus1	841	-12	-64	-26	-5.2
Right middle cingulate gyrus	150	6	12	38	4.8
Right superior frontal gyrus	209	10	32	44	3.7
Right thalamus	450	2	-24	4	-5.29
Right inferior temporal gyrus	1190	36	-58	-24	-6.58
**Test 3 vs Test 1**					
Left middle temporal gyrus	105	-60	-30	-2	-4.46
Left lingual gyrus	148	-22	-64	-18	-3.50
Left cerebelum_crus1	266	-40	-60	-30	-5.54
Left parietal gyrus	266	-42	-62	32	-5.29
Left middle temporal gyrus	339	-40	-36	-8	-4.69
Left cingulate gyrus	515	-4	34	2	-5.74
Right amygdala	109	30	0	-24	-4.68
Right hippocampus	127	38	-28	-8	-4.27
Right fusiform gyrus	303	32	-66	-16	-4.24
Right cerebellum	2464	2	-44	-34	-6.30

Appendix

Informed consent form

Dear volunteers:

We will invite you to participate in a study on "The Adaptation Mechanisms of the Brain in High Altitude Environments". This study has been approved by the ethics committee of the Medical College of Xiamen University. Before you take the test, please read the following carefully so that you can understand the purpose and content of the test, as well as the benefits and risks that may be brought to you. You can discuss it with your teachers and classmates to help you decide whether to take this science test.

1. The purpose of this project is to understand the adaptation mechanism of the brain in the high-altitude environment.
2. All subjects are required to have a normal body mass index, be non-smokers, and be right-handed. Subjects with a past history of neurological disorders, head injuries, and psychiatric disorders will be excluded.
3. The test contents include blood routine examination, cognitive behavior, and brain magnetic resonance examination (about magnetic resonance examination, we will explain it to you in another informed consent form).

Benefits of participating in the project: we will provide you with blood routine examination, cognitive examination, brain structure, and brain function examination free of charge. You will get some economic rewards after the test.

Risk of participating in the project: participating in the project will not do any harm to your health.

Confidentiality principle: we will keep all your information (basic personal information, screening results) confidential for you.

Voluntary principle: whether you participate in this project is entirely voluntary. You can exit the test at any time for any reason. If you decide not to participate in the test or you want to withdraw from the test, it will not cause any damage to your original interests and you will not have to pay any fee. This test is free.

We sincerely look forward to your participation!

Consent statement: I have read this informed consent form and understand all relevant

situations. I decide to agree to participate in this study and cooperate as much as possible.

Name:________________________________

Telephone number: ____________________

Date: _____________________

Informed consent form of magnetic resonance imaging

Dear volunteers:

Thank you for completing this test with us. This test uses magnetic resonance imaging to observe the adaptation mechanism of the brain in high altitude environment. Magnetic resonance is a non-invasive medical examination method developed in the 1990s. This method uses the external magnetic field to image the human structure and tissue. During the examination, the subject will not contact any radioactive substances such as radiation sources and isotopes, and does not have to take or inject any drugs. Since the application of MRI in the clinic, there has been no maladjustment caused by this test method so far. You can accept the examination at ease.

1. Before you are enrolled in this study, the doctor will ask and record your medical history, and conduct a physical examination and a simple behavioral test. If you are selected, you can participate as you wish and sign the informed consent form.

2. Before entering the MRI room, please cooperate with the operator to check the following contents:

(yes, no) carry metal items (keys, watches, etc.);

(yes, no) carry magnetic items (magnetic card, IC card, etc.);

(yes, no) wear metal accessories (brooch, hairpin, etc.);

(yes, no) there are metal parts on the clothing (belt buckle, metal zipper, etc.);

(yes, no) any surgery (what kind of surgery:);

(yes, no) there may be metal objects in the body (metal denture, orthodontic appliance, bone fixation plate, aneurysm clip, shrapnel, birth control ring, etc.);

(yes, no) use medical equipment (pacemaker, etc.) in the body.

3. The research unit and main researchers shall bear the following responsibilities:

1) The data and personal data obtained in the whole study are only used for scientific research, not for any news and commercial purposes;
2) Strictly keep the personal data involved confidential;
3) Show this informed consent before the study, and remove the requirements and precautions for examination;
4) After the research is completed, a certain economic reward will be given at one time;
5) In principle, MRI films will not be provided free of charge, and the scanned original documents will be restricted after negotiation between both parties;
6) If abnormal brain structure is found in the scan, medical advice will be given free of charge, but no further diagnosis and treatment will be provided.

4. Volunteers participating in the study should provide the following guarantees:

1) Participate in this study knowingly and voluntarily, cooperate with all research requirements, and truthfully answer questions;
2) The ownership and use right of all data obtained from the research belongs to the unit implementing the research;
3) After obtaining one-time economic remuneration, no other economic requirements shall be added.

5. Volunteers participating in the study shall also have the following rights:

1) During the study, all MRI scans were received free of charge;
2) When the requirements for MRI scanning and examination are unclear, you can ask;
3) You can view your electronic data of brain anatomical structure images for free;
4) Timely put forward opinions and suggestions on aspects that are dissatisfied and uncomfortable in the research process.

I have read the above agreement and agree, volunteer:_________________

I have read the above agreement and agree, researcher:_________________

I have read the above agreement and agree, imaging operator:____________
